# Pro and Anti-Tobacco Messaging Exposure in a Nationally Representative Sample of U.S. Older Adults

**DOI:** 10.1093/geroni/igaf122.2513

**Published:** 2025-12-31

**Authors:** Margaret Fahey, Siyuan Huang

**Affiliations:** Middle Tennessee State University, Murfreesboro, Tennessee, United States; University of Arizona, Tucson, Arizona, United States

## Abstract

Adults are more impacted by the negative health consequences of tobacco use as they age, yet cigarette prevalence rates have stagnated among U.S. adults 65+ years for the past 15 years. Despite the harm reduction potential of switching from cigarettes to e-cigarettes, older adults are more likely to misperceive the relative harms of these products. With changing patterns of media consumption, updated information is needed regarding the platforms in which this age group receives tobacco messaging. Using U.S. nationally representative data from the 2022 Health Information and National Trends Survey, we measured pro- and anti-tobacco messaging exposure among adults 65+ years via internet (television/streaming, social media, websites) and non-internet (stores, billboards, pharmacies, bars/restaurants, events, radio, print) platforms. Secondarily, we assessed main effects and interactions of age (65+ vs. < 65 years) and cigarette smoking in relation to tobacco messaging platforms and harm beliefs. Pro-tobacco messaging mostly occurred via stores (46%), television/streaming (11%), and billboards (9%) among older adults who smoke cigarettes. Anti-tobacco messaging mostly occurred via television/streaming (53%), stores (28%), and print media (25%). Older (vs. younger) adults were less likely to receive pro-tobacco messaging across internet and non-internet platforms (p’s<.03) and less likely to receive anti-tobacco messaging via non-internet platforms (p<.001). No age differences were found for anti-tobacco messaging via internet platforms (p>.72). Finally, older adults were more likely to report not knowing about relative e-cigarette harms (p<.001). Among this high priority population, television and streaming platforms might be a modality in which to disseminate tobacco harm reduction education.

